# Dose-expansion study of OBI-999, a Globo H-targeting antibody-drug conjugate, in patients with advanced solid tumors

**DOI:** 10.1093/oncolo/oyag249

**Published:** 2026-06-29

**Authors:** Apostolia Maria Tsimberidou, Darren Sigal, Anna Varghese, Daniel Vaena, Li-Yuan Bai, Ming-Huang Chen, Chun-Nan Yeh, Chia-Jui Yen, Mehmet A Baysal, Abhijit Chakraborty, Ingly Lee, Chi-Sheng Shia, Konul Ristoski, Dong Xu

**Affiliations:** Department of Investigational Cancer Therapeutics, The University of Texas MD Anderson Cancer Center, Houston, TX 77030, United States; Scripps Clinic and Scripps Cancer Center, La Jolla, CA 92037, United States; Memorial Sloan Kettering Cancer Center, New York, NY 10022, United States; West Cancer Center & Research Institute, Germantown, TN 38138, United States; China Medical University Hospital, Taichung, Taichung City 404, Taiwan; Taipei Veteran General Hospital, Taipei, Taipei City 11217, Taiwan; Chang Gung Memorial Hospital, Taipei City, Taoyuan City 333, Taiwan; Department of Oncology, National Cheng Kung University Hospital, College of Medicine, National Cheng Kung University, Tainan, Tainan City 70403, Taiwan; Department of Investigational Cancer Therapeutics, The University of Texas MD Anderson Cancer Center, Houston, TX 77030, United States; Department of Investigational Cancer Therapeutics, The University of Texas MD Anderson Cancer Center, Houston, TX 77030, United States; OBI Pharma Inc., Taipei City 115, Taiwan; OBI Pharma Inc., Taipei City 115, Taiwan; OBI Pharma USA, Inc., San Diego, CA 92121, United States; OBI Pharma USA, Inc., San Diego, CA 92121, United States

**Keywords:** antibody-drug conjugate, basket trial, Globo H, immunotherapy, MMAE, precision medicine, solid tumors, translational research

## Abstract

**Background:**

OBI-999 is an antibody-drug conjugate consisting of the Globo H-targeting antibody OBI-888 linked to the cytotoxic payload monomethyl auristatin E. In a dose-escalation study, the OBI-999 recommended phase 2 dose (RP2D) was 1.2 mg/kg every 3 weeks. This dose-expansion study evaluated the efficacy and safety of OBI-999 in patients with pancreatic cancer, colorectal cancer (CRC), or other tumor types (“basket cohort”) and tumoral Globo H H-scores ≥100 (NCT04084366).

**Patients and Methods:**

Patients were treated at RP2D of OBI-999. We assessed response, progression-free survival (PFS), treatment-emergent adverse events (TEAEs), and pharmacokinetics of OBI-999.

**Results:**

Of 29 patients treated, 23 were evaluable for response. Eight (34.8%) had stable disease): pancreatic cancer, 4 of 7; CRC, 2 of 8; basket cohort, 2 of 8. No objective response was noted. The median time on treatment was 22 days. No association between H-score and tumor size reduction was noted. Median PFS was 3.3 months, 1.3 months, and 1.4 months in the pancreatic, CRC, and basket cohorts, respectively. One patient (3.4%) experienced a serious TEAE related to OBI-999 (febrile neutropenia) that led to discontinuation of OBI-999; the TEAE resolved 3 days after onset. OBI‑999 showed consistent pharmacokinetics with comparable exposure at RP2D across cohorts.

**Conclusions:**

OBI-999 had a favorable safety profile. Although 34.8% of patients had disease stabilization, no objective response was noted, likely owing to inefficient antigen binding, suboptimal systemic exposure at the tolerated dose, and complex tumor biology.

Lessons learned

- This study provides the first dose-expansion clinical evaluation of OBI-999 in patients with advanced solid tumors, including pancreatic and colorectal cancers. OBI-999 demonstrated a favorable safety profile but limited efficacy.
- Although 34.8% of patients had disease stabilization, no objective response was noted, likely owing to inefficient antigen binding and internalization, suboptimal systemic exposure at the tolerated dose, and complex tumor biology.
- These findings highlight the challenges of targeting Globo H with a single-agent antibody-drug conjugate (ADC) and suggest that future investigations may require combination strategies, improved ADC design, or optimized patient selection based on tumor biology.

## Trial information

**Table:** 

**Disease**	Advanced solid tumors
**Stage of disease/treatment**	Advanced, metastatic/OBI-999, a Globo H-targeting antibody-drug conjugate
**Prior therapy**	Many lines, standard of care
**Type of study**	Expansion
**Primary endpoints**	Response, progression-free survival
**Secondary endpoints**	Safety, including treatment-emergent adverse events, and the pharmacokinetic parameters of OBI-999 and its active metabolite
**Additional details of endpoints or study design** *Patients* Patients ≥18 years old who had histologically or cytologically confirmed advanced solid tumors for which previous standard-of-care therapy was deemed no longer effective or who declined to receive further standard-of-care treatments were eligible. Patients were required to have a documented Globo H H-score ≥100 (on a 0-300 scale) determined by a qualified laboratory immunohistochemical assay, as previously described,^1^^,^^2^ in one of the following tumor types: pancreatic cancer, colorectal cancer, and other solid tumor types (Figure 1A). The assay was developed and implemented by NeoGenomics Laboratories (San Diego, CA, USA) and authorized by the FDA for clinical study use.^1^^,^^2^ Patient screening based on an H-score ≥100, indicative of moderate to high Globo H overexpression, was part of the FDA authorization.^3^ Detailed eligibility criteria are shown in Supplementary Material. This study was conducted at 8 centers (4 in the United States, 4 in Taiwan) and was approved by each independent ethics committee or institutional review board. The study was conducted in accordance with ethical principles of the International Council on Harmonization Guideline for Good Clinical Practice, the Declaration of Helsinki, and applicable local regulations. All patients provided written informed consent stating that they were aware of the investigational nature of the study. *Study design* This was a phase 1/2, open-label, dose-escalation, and dose-expansion study. Patients were enrolled in Part B (dose expansion) using Simon’s 2-stage cohort expansion design. Part B was conducted to assess the preliminary efficacy of OBI-999 in Globo H-expressing advanced solid tumors, to provide additional safety data, and to characterize the pharmacokinetic (PK) profile of OBI-999. *Treatment* Patients received the recommended phase 2 dose (RP2D) dose of 1.2 mg/kg OBI-999 (capping calculations at a maximum weight of 100 kg) as an intravenous (IV) infusion on day 1 of each 21-day cycle. The infusion was administered for a duration of 60 minutes (± 10 minutes) for the initial 2 cycles, with subsequent cycle durations reduced to approximately 30 minutes at the investigator’s discretion, if no infusion-related adverse events occurred during the first 2 cycles. All patients received pre-medications 30-60 minutes prior to OBI-999 administration, including acetaminophen and diphenhydramine. Patients could continue to receive treatment with OBI-999 until disease progression, unacceptable toxicity, consent withdrawal, or up to 35 cycles (approximately 2 years), whichever occurred first. Imaging studies (computed tomography or magnetic resonance imaging) for response assessment were performed at baseline, every 6 weeks for the first 3 months, and then every 9 weeks thereafter. The safety follow-up visit was conducted 28 days after the last dose of OBI-999. *Endpoints and statistical analysis* The primary endpoint was the objective response rate (complete response [CR] and partial response [PR]), using RECIST v1.1.^4^ We also assessed the clinical benefit rate (CR + PR + stable disease [SD]), and progression-free survival (PFS). A confirmed response was defined as 2 or more consecutive assessments separated by at least 3 weeks. The percentage of patients with anti-drug antibodies against OBI-999 (ADAs) in their blood was another protocol-specified primary endpoint. Details are described in Supplementary Material. Secondary endpoints included safety evaluations (treatment-emergent adverse events [TEAEs], serious TEAEs, and laboratory abnormalities) and PK parameters for total antibody, OBI-999, and its active metabolite monomethyl auristatin E (MMAE). Details are described in Supplementary Material. The safety population comprised all enrolled patients who received at least 1 dose of OBI-999. The efficacy population included all enrolled patients who received at least 1 dose and had at least 1 follow-up tumor assessment scan. Following a protocol amendment, intensive pharmacokinetic sampling was performed only for the first 5 patients in each cohort. This dose-expansion phase of the trial was designed to enroll up to 57 patients, with the first stage designed to recruit up to 9 patients in each of 3 cohorts. If at least 1 objective response was observed, a second stage of recruitment would proceed, enrolling up to 10 additional patients in the corresponding cohort, for a total of up to 19 patients per cohort. The study was designed to follow patients up to a 24-week scheduled response assessment to evaluate objective response rates and determine Simon’s 2-stage design success criteria. The sample size was based on a one-sided alpha of 0.12 and 72% power. Descriptive statistics were generated for continuous variables, and descriptive summaries of time-to-event data included medians and confidence intervals. Kaplan–Meier estimates and 95% confidence intervals were calculated for PFS.	

## Drug information

Globo H, a glycosphingolipid, has been considered a promising therapeutic target for cancer immunotherapy, given its lack of expression in normal tissues coupled with its overexpression on the cell surface of common cancers of epithelial origin, including pancreatic, gastric, lung, colorectal, esophageal, gallbladder, and breast cancers.^3^^,^^5–8^ Higher expression of Globo H is correlated with more aggressive tumors and poorer clinical outcomes, and its role in tumor biology appears to be in part through promotion of angiogenesis and potential suppression of immune cell activity.^6–10^

**Figure 1.**
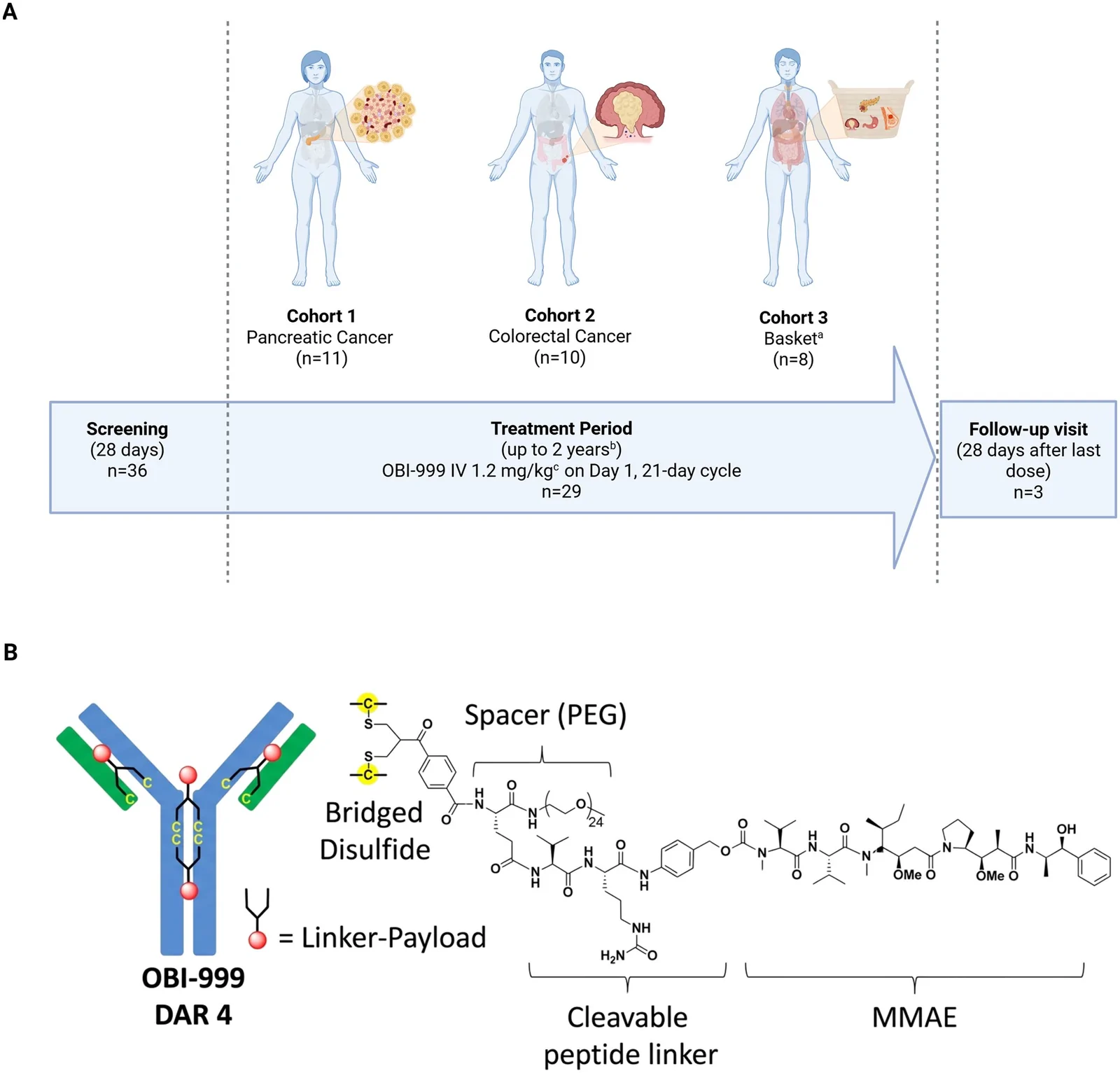
OBI-999 structure and phase 2 trial design. (A) Study design for the Part B dose-expansion phase of the OBI-999 trial. (B) Structure of the antibody-drug conjugate OBI-999, composed of a Globo H–targeting monoclonal antibody (OBI-888) conjugated to monomethyl auristatin E (MMAE) via a ThioBridge valine-citrulline-p-aminobenzyloxycarbonyl linker. ^a^Any solid tumor types other than those in Cohorts 1 and 2. ^b^Patients received treatment until disease progression, unacceptable toxicity, consent withdrawal, or for up to 35 cycles (approximately 2 years), whichever occurred first. ^c^The administered dose was capped at a maximum of 120 mg for patients of 100 kg and above. Panel A adapted from Tsimberidou et al.,^1^ licensed under a Creative Commons Attribution Noncommercial-NoDerivatives 4.0 International License (CC BY-NC-ND 4.0). Panel B created with BioRender.com. Abbreviations: DAR, drug-to-antibody ratio; IV, intravenous; MMAE, monomethyl auristatin E; PEG, polyethylene glycol.

Clinical development efforts for Globo H-targeting therapies include antibodies, antibody-drug conjugates (ADCs), vaccines, and cell therapy using chimeric antigen receptor T (CAR T) cells.^1^^,^^2^^,^^11^^,^^12^ OBI-999 is an ADC that combines a Globo H-targeting antibody (OBI-888) with the cytotoxic agent MMAE via a ThioBridge conjugate valine-citrulline-p-aminobenzyloxycarbonyl linker (Figure 1B).^1^ OBI-999 binds to Globo H on cancer cells and is internalized and trafficked to lysosomes, where the linker is cleaved by cathepsin B, leading to the release of MMAE and suspension of the cell cycle.^1^^,^^13^^,^^14^ OBI-999 received an orphan drug designation from the US FDA to target pancreatic cancer (2019) and gastric cancer (2020).^15^^,^^16^

**Figure 2.**
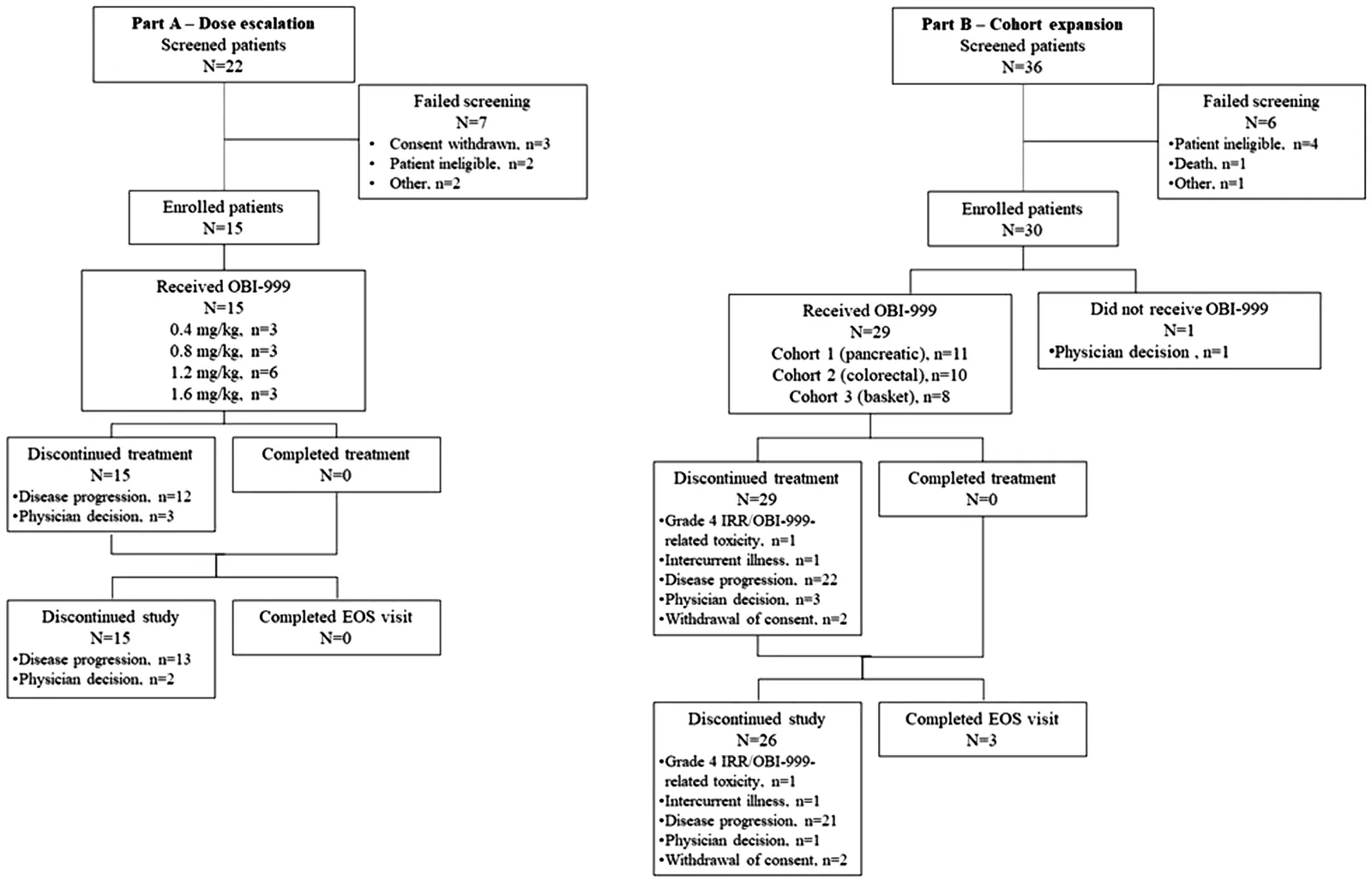
Consort diagram. Patients who underwent screening for treatment and were treated with OBI-999.

**Figure 3.**
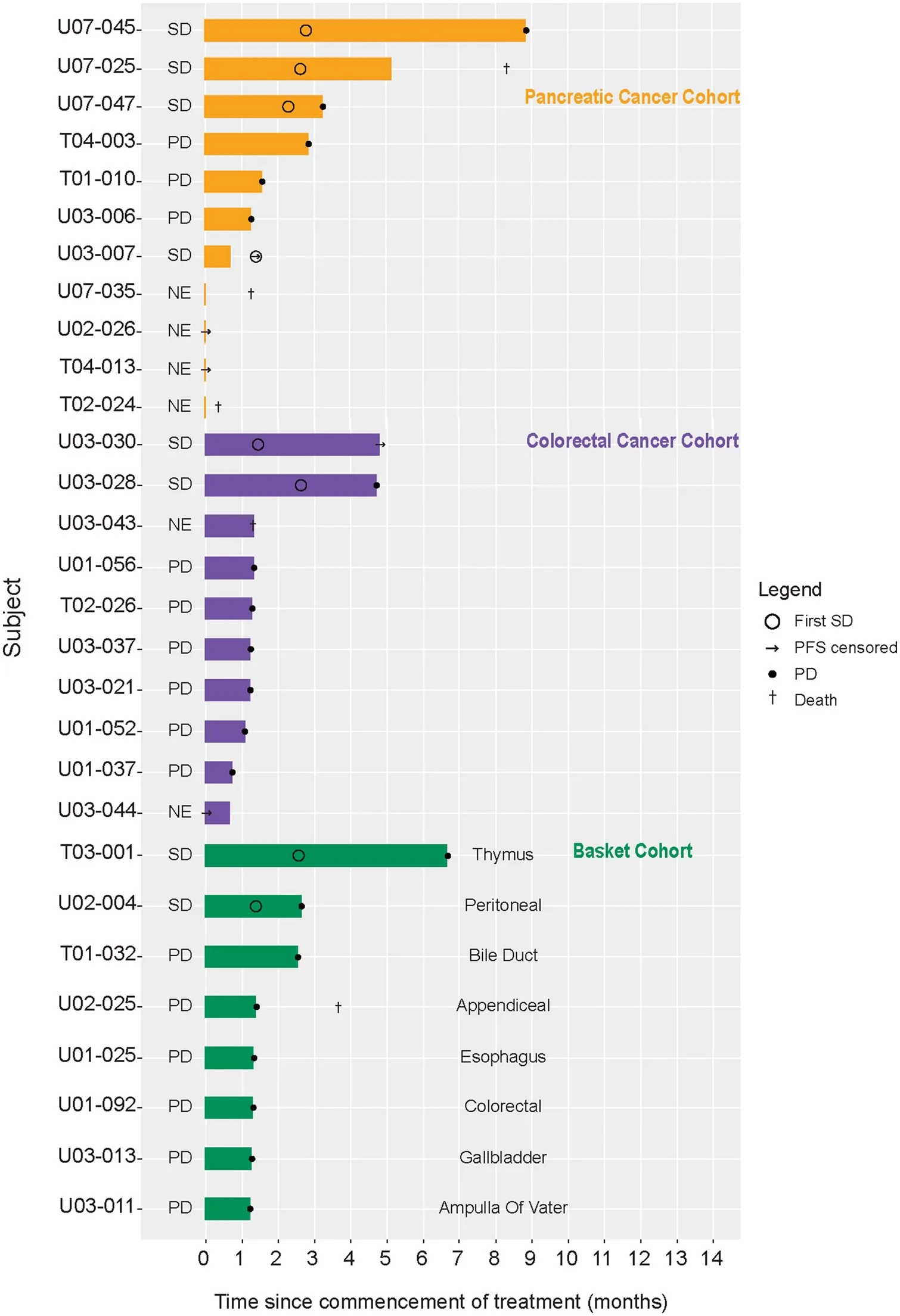
Time on treatment and disease course by patient. Swimmer plot showing the duration of OBI-999 treatment, treatment response, and progression-free survival (PFS) for each patient. ^a^Each bar represents a patient; bar length corresponds to duration on treatment. Markers indicate best overall response per RECIST v1.1. Patients with stable disease (SD), progressive disease (PD), and not evaluable (NE) are shown across the 3 cohorts (pancreatic cancer, colorectal cancer, and basket). ^a^All patients who were on the study for only 1 day received OBI-999 with no interruption or premature discontinuation of infusion. Of the 4 patients who received OBI-999 during the study and subsequently died, 2 deaths were attributed to disease progression, and 2 deaths were due to adverse events (sepsis, septic shock) that were deemed unrelated to OBI-999. Patient U03-007 had clinical progression 22 days after initiation of treatment and stable disease by RECIST on day 43. Patient U03-030 had clinical progression 129 days after initiation of treatment and stable disease by RECIST on day 147.

**Figure 4.**
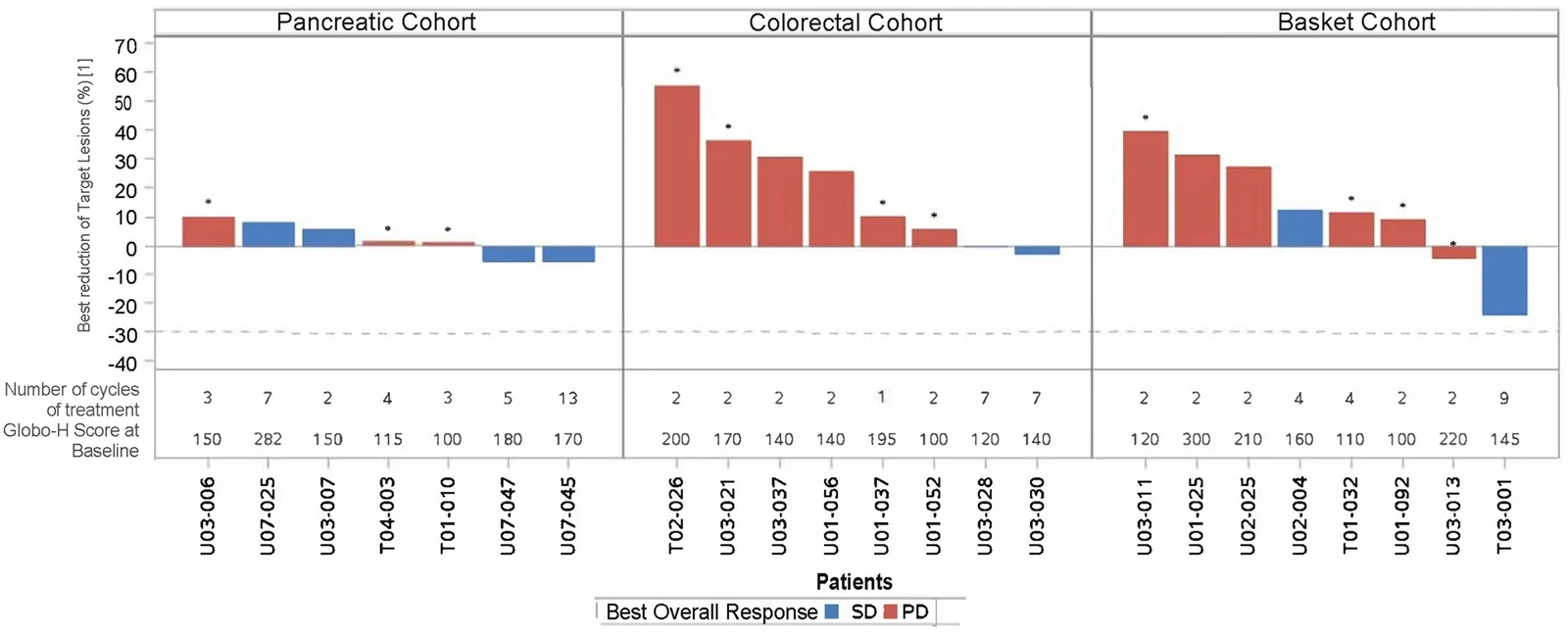
Maximum percent change in tumor size from baseline in evaluable patients. Waterfall plot showing maximum percent change in target lesion size from baseline for each evaluable patient. Dotted line at ≥30% denotes the RECIST v1.1 threshold for partial response. Bars above 0% indicate tumor growth; bars below 0% indicate tumor reduction. Patients with new lesions are marked with an asterisk (*). For each patient, best overall response (CR, PR, SD, PD, or NE) was measured from the start of treatment until disease progression, recurrence, or end of study. In general, the patient’s best overall response depended on both the tumor measurement and confirmation criteria. ^1^The maximum negative percentage change from baseline for each patient with at least one reduction or the minimum positive percentage change from baseline for each patient without any reduction. Abbreviations: PD, progressive disease; SD, stable disease.

**Figure 5.**
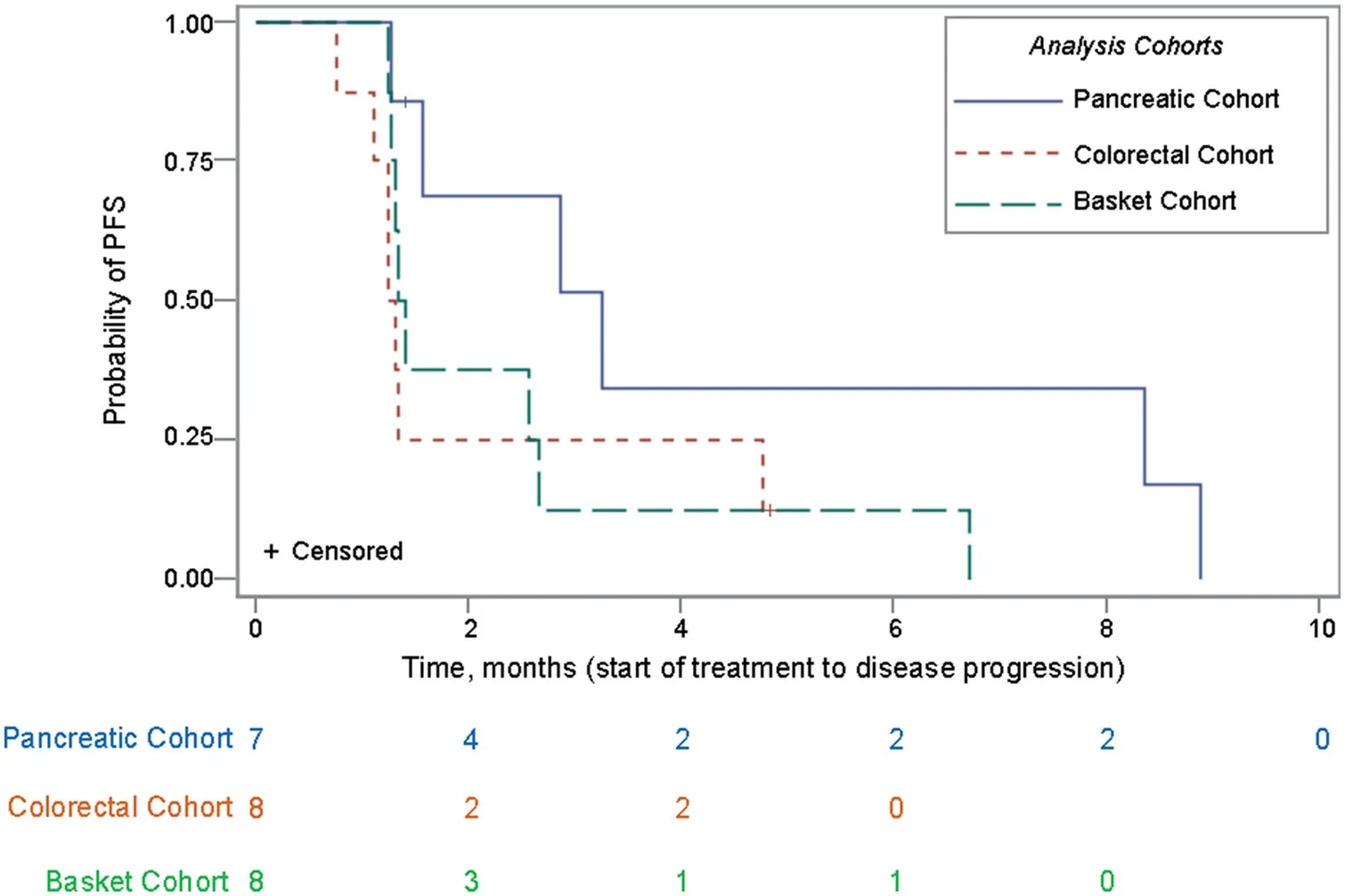
Kaplan–Meier curves for progression-free survival (PFS). PFS curves by tumor cohort (pancreatic, colorectal, basket) for patients treated with OBI-999. Median PFS and 95% confidence intervals are indicated for each cohort. PFS was defined as time from first dose to disease progression or death from any cause.

A phase 1/2, first-in-human, open-label, dose-escalation (Part A) and dose-expansion (Part B) study of OBI-999 was conducted in patients with advanced solid tumors (NCT04084366). We previously reported results from 22 patients with advanced solid tumors enrolled in Part A.^1^ In that sequential dose-escalation group, the maximum tolerated dose (MTD) and RP2D of OBI-999 was established as 1.2 mg/kg.^1^

Here, we report the results of the OBI-999 dose-expansion study (Part B). The combination of unmet need, impact potential, and Globo H overexpression (50% of pancreatic and 16% of colorectal tumors^3^) that enabled biomarker-driven patient selection led to the inclusion of 3 cohorts in Part B: pancreatic cancer, colorectal cancer, and other tumor types (basket cohort). The primary endpoints were the objective response rate and PFS, in patients with Globo H-expressing pancreatic, colorectal, and other tumors. The secondary endpoints were safety, including TEAEs, and the PK parameters of OBI-999 and its active metabolite MMAE.

**Table:** 

**DRUG INFORMATION**	
**Generic/working name**	OBI-999
**Company name**	OBI Pharmaceuticals
**Drug type**	Antibody drug conjugate
**Drug class**	Targeted therapy
**Dose**	1.2 mg/kg
**Route**	IV
**Schedule of administration**	Every 3 weeks

**Table:** 

EXPANSION COHORTS			
**Tumor types**	**Dose of drug**	**Number enrolled**	**Number evaluable for toxicity**
**Pancreatic cancer**	1.2 mg/kg	11	11
**Colorectal cancer**	1.2 mg/kg	10	10
**Various (basket)**	1.2 mg/kg	8	8

## Patient characteristics

**Table:** 

	Pancreatic cancer cohort (*n* = 11)	Colorectal cancer cohort (*n* = 10)	Basket cohort^a^ (*n* = 8)	Total (*N* = 29)
**Age, mean (STDEV), years**	62.1 (11.5)	59.6 (10.7)	62.9 (8.2)	61.4 (10.1)
**Age, median (range), years**	63.0 (43-79)	60.5 (40-77)	65.5 (49-74)	63.0 (40-79)
**Male, *n* (%)**	6 (54.5)	5 (50.0)	4 (50.0)	15 (51.7)
**Ethnicity, *n* (%)**				
** Hispanic or Latino**	0	1 (10.0)	0	1 (3.4)
** East Asian**	4 (36.4)	1 (10.0)	2 (25.0)	7 (24.1)
** Other**	7 (63.6)	6 (60.0)	6 (75.0)	19 (65.5)
** Unknown**	0	2 (20.0)	0	2 (6.9)
**Race, *n* (%)**				
** White**	7 (63.6)	5 (50.0)	5 (62.5)	17 (58.6)
** Asian**	4 (36.4)	2 (20.0)	3 (37.5)	9 (31.0)
** Other**	0	2 (20.0)	0	2 (6.9)
** Unknown**	0	1 (10.0)	0	1 (3.4)
**Body mass index, mean (STDEV), kg/m^2^**	23.0 (3.9)	24.3 (4.3)	26.4 (10.0)	24.4 (6.2)
**ECOG performance status, *n* (%)^b^**				
** 0**	4 (36.4)	1 (10.0)	2 (25.0)	7 (24.1)
** 1**	7 (63.6)	8 (80.0)	6 (75.0)	21 (72.4)
**Stage, *n* (%)**				
** 2**	2 (18.2)	0	1 (12.5)	3 (10.3)
** 3**	1 (9.1)	1 (10.0)	0	2 (6.9)
** 4**	8 (72.7)	8 (80.0)	6 (75.0)	22 (75.9)
** Unknown**	0	0	1 (12.5)	1 (3.4)
**Globo H H-Score, mean (STDEV)**	176.1 (53.7)	143.5 (35.1)	170.6 (68.4)	163.3 (53.1)
**Globo H H-Score, median (range)**	170.0 (100-282)	140.0 (100-200)	152.5 (100-300)	150.0 (100-300)

a Tumor types in the basket cohort were colorectal (*n* = 1), esophageal (*n* = 1), appendiceal (*n* = 1), gallbladder (*n* = 1), ampulla of Vater (*n* = 1), peritoneal (*n* = 1), bile duct (*n* = 1), thymus (*n* = 1). The patient with colorectal cancer was enrolled in the basket cohort rather than the colorectal cohort because the colorectal cohort had reached its target accrual by the time the patient provided written informed consent.

b One patient in the colorectal cancer cohort had a missing ECOG score at baseline; when assessed at cycle 3, ECOG was 1.

Abbreviations: ECOG, Eastern Cooperative Oncology Group; STDEV, standard deviation.

**Table:** 

**PRIMARY ASSESSMENT METHOD**	
**Title**	
**Number of patients screened**	36
**Number of patients enrolled**	29
**Number of patients evaluable for toxicity**	29
**Number of patients evaluated for efficacy**	23
**Evaluation method**	RECIST 1.1
**Outcome Notes**: From November 25, 2019, to October 27, 2023, 36 patients were screened, and 29 received OBI-999 treatment. Of the 7 patients who did not receive treatment, 5 were deemed ineligible, 1 died, and 1 was removed from the trial by the physician’s decision (Figure 2). The basket cohort permitted enrollment of patients with either pancreatic or colorectal cancer. However, 1 patient with colorectal cancer was enrolled in the basket cohort because due to competitive enrollment, the colorectal cohort had reached its target accrual by the time the patient signed the informed consent document and therefore the patient was permitted to be enrolled in the basket cohort. The median age was 63 years (range, 40-79 years), and approximately equal proportions of men and women were treated with OBI-999. Patients were predominantly White (17/29, 58.6%) and approximately one-third were Asian (9/29, 31.0%). The median Globo H H-score was 150 (range, 100-300). The median number of OBI-999 doses was 2 (range, 1-13), with 82.8% (24/29) of patients receiving ≥2 doses. The median total administered dose of OBI-999 was 157 mg (range, 65-762 mg), and the median time on treatment was 22 days (range, 1-254 days) (Table 1). A total of 7 patients had received nab-paclitaxel or docetaxel treatment, including 3 patients with pancreatic cohort, 1 patient with colorectal cohort, and 3 patients in the basket cohort. Among them, there were 3 (100%), 1 (100%), and 3 (100%) patients who had PD respectively. Twenty-three patients (7 of 11 pancreatic, 8 of 10 colorectal, 8 of 8 basket) were evaluable for response (i.e., received ≥1 dose of study drug and had ≥1 follow-up tumor assessment scan). The time on OBI-999 treatment and disease course for each patient are shown in Figure 3. No objective response was noted across the 3 cohorts. The best overall response was SD, and was observed in 8 (8/23, 34.8%) patients. The clinical benefit rate (CR + PR + SD) was therefore also 34.8% (8/23 patients). Overall, 4 of 7 evaluable patients in the pancreatic cancer cohort, 2 of 8 patients in the colorectal cancer cohort, and 2 of 8 patients in the basket cohort (1 with cancer of the thymus and 1 with peritoneal cancer) had SD. The remaining patients had progressive disease as their best response (Figure 3). The waterfall plot showing the best percent reduction in target lesions across the 3 cohorts is illustrated in Figure 4. The patient with the greatest reduction in tumor size (23.5%) was a patient with cancer of the thymus (basket cohort). No clear relationship between baseline Globo H score and percent tumor size reduction was observed in any of the cohorts (Figure 4). The median PFS was 3.3 months (95% CI: 1.28-NE months) in the pancreatic cancer cohort, 1.3 months (95% CI: 0.76-4.76 months) in the colorectal cancer cohort, and 1.4 months (95% CI: 1.25-2.66 months) in the basket cohort (Figure 5). All patients discontinued treatment. The primary reason for treatment discontinuation was disease progression, reported in 22 of 29 (75.9%) patients (Figure 2). Additionally, based on the statistical plan, each cohort would enroll 9 patients. If 1 response was noted, an additional 10 patients would be enrolled. However, the pancreatic and colorectal cancer cohorts enrolled more than 9 patients, although there were no objective responses. More than 9 patients were enrolled because competitive recruitment could not fully control the enrollment rate at each site. This may lead to imbalanced enrollment across sites. However, our focus was to ensure that the overall enrollment remained as close as possible to the planned number.	

## Treatment-emergent adverse events in patients treated with OBI-999

**Table:** 

	Patients, *n* (%)			
	Pancreatic Cancer Cohort (*n* = 11)	Colorectal Cancer Cohort (*n* = 10)	Basket Cohort (*n* = 8)	Total (*N* = 29)
**TEAEs**	11 (100)	10 (100)	7 (87.5)	28 (96.6)
**Related^a^**	8 (72.7)	6 (60.0)	6 (75.0)	20 (69.0)
**TEAEs grade ≥3**	7 (63.6)	5 (50.0)	0	12 (41.4)
**Serious TEAEs**	8 (72.7)	2 (20.0)	0	10 (34.5)
**Related ^a^**	1 (9.1)	0	0	1 (3.4)
**TEAE leading to treatment discontinuation**	1 (9.1)^b^	0	0	1 (3.4)
**TEAE leading to death**	1 (9.1)^c^	1 (10.0)^d^	0	2 (6.9)
**Most common treatment-related TEAEs (≥ 2% in any cohort)^a^**				
**Fatigue**	6 (54.5)	2 (20.0)	2 (25.0)	10 (34.5)
**Myalgia**	2 (18.2)	2 (20.0)	0	4 (13.8)
**Diarrhea**	3 (27.3)	1 (10.0)	0	4 (13.8)
**Muscle spasms**	2 (18.2)	0	1 (12.5)	3 (10.3)
**Decreased appetite**	2 (18.2)	0	1 (12.5)	3 (10.3)
**Decreased platelet count**	0	0	2 (25.0)	2 (6.9)
**Electrocardiogram QT prolonged**	1 (9.1)	1 (10.0)	0	2 (6.9)
**Decreased weight**	1 (9.1)	0	1 (12.5)	2 (6.9)
**Arthralgia**	0	2 (20.0)	0	2 (6.9)
**Nausea**	1 (9.1)	0	1 (12.5)	2 (6.9)
**Vomiting**	0	1 (10.0)	1 (12.5)	2 (6.9)
**Pruritus**	1 (9.1)	0	1 (12.5)	2 (6.9)

a Related events are defined as those reported as “possibly related,” “probably related,” or “definitely related” or recorded without relationship information.

b TEAE leading to treatment discontinuation was febrile neutropenia; the event resolved, and the patient recovered from the event.

c TEAE leading to death was sepsis.

d TEAE leading to death was septic shock.

Abbreviation: TEAE, treatment-emergent adverse event.

## Outcome notes

### Safety

All patients who received OBI-999 were assessed for safety. Overall, 96.6% of patients reported TEAEs in Part B. Of 29 patients, 1 (3.4%) reported a serious TEAE related to OBI-999 (febrile neutropenia) that led to study discontinuation; the TEAE resolved, and the patient recovered 3 days after onset. The most frequently occurring treatment-related AEs were fatigue (34.5%), myalgia (13.8%), and diarrhea (13.8%). The percentage of patients who experienced TEAEs related to OBI-999 was 72.7% in the pancreatic cancer cohort, 60.0% in the colorectal cancer cohort, and 75.0% in the basket cohort. Four patients with pancreatic carcinoma and 2 with colorectal cancer experienced ≥grade 3 treatment-related AEs, including febrile neutropenia, decreased neutrophil count, fatigue, increased blood bilirubin, and increased aspartate aminotransferase (Table 2). The only grade ≥ 3 TEAE that was reported in more than 2 patients was fatigue (4/29 patients, 13.8%).

**Table 1.** Doses and time on treatment.

	Cohort 1	Cohort 2	Cohort 3	Total (*N* = 29)
	Pancreatic cancer (*n* = 11)	Colorectal cancer (*n* = 10)	Basket (*n* = 8)	
**Total number of doses received—categorical, *n* (%)**				
** 1**	4 (36.4)	1 (10.0)	0	5 (17.2)
** 2**	1 (9.1)	7 (70.0)	5 (62.5)	13 (44.8)
** 3**	2 (18.2)	0	0	2 (6.9)
** 4**	1 (9.1)	0	2 (25.0)	3 (10.3)
** 5**	1 (9.1)	0	0	1 (3.4)
** 7**	1 (9.1)	2 (20.0)	0	3 (10.3)
** 9**	0	0	1 (12.5)	1 (3.4)
** 13**	1 (9.1)	0	0	1 (3.4)
**Total number of doses received—continuous**				
**Mean (STDEV)**	3.7 (3.6)	2.9 (2.2)	3.4 (2.5)	3.3 (2.8)
**Median (min–max)**	3.0 (1-13)	2.0 (1-7)	2.0 (2-9)	2.0 (1-13)
**Total actual dose received (mg)**				
**Mean (STDEV)**	244.4 (187.3)	247.1 (238.3)	263.3 (173.1)	250.6 (195.8)
**Median (min–max)**	165.0 (65.0-680.0)	153.1 (79.5-762.1)	171.3 (126.0-566.0)	156.8 (65.0-762.1)
**Time on treatment (days)**				
**Mean (STDEV)**	62.5 (79.9)	41.4 (46.6)	53.6 (58.1)	52.8 (62.5)
**Median (min–max)**	43.0 (1-254)	22.0 (1-129)	22.5 (21-189)	22.0 (1-254)

Abbreviation: STDEV, standard deviation.

**Table 2.** Summary of grade ≥3 treatment-related adverse events.

Patient ID	Cohort	Adverse event preferred term	Day on study	Grade	SAE	Relationship to the drug	Resolved
**T04-013**	Pancreatic	Febrile neutropenia	15	4	Yes	Possible	Yes
**U03-006**	Pancreatic	Neutrophil count decreased	37	4	No	Probable	Yes
		Fatigue	61	3	No	Probable	No
**U03-007**	Pancreatic	Fatigue	46	3	No	Possible	No
**U03-021**	Colorectal	Fatigue	28	3	No	Possible	No
**U03-043**	Colorectal	Blood bilirubin increased	16	3	No	Possible	No
**U07-035**	Pancreatic	Aspartate aminotransferase increased	8	3	No	Possible	Yes

Abbreviations: ID, identification number; SAE, serious adverse event.

Two deaths due to TEAEs were reported during the clinical trial, neither of which was attributed to OBI-999. A man in his 60s with pancreatic cancer who was diagnosed with COVID-19, 15 days prior to the initiation of the study drug, was hospitalized for shortness of breath and productive cough on cycle 1, day 4. A chest X-ray demonstrated pneumonia and right-sided pleural effusion. He was started on supplemental oxygen. The white blood cell count (WBC) was 13.8 × 10^9^/L (absolute neutrophil count [ANC] = 10.05 × 10^9^/L). *Pneumocystis jiroveci* was identified in the sputum. He was treated with sulfamethoxazole/trimethoprim 960 mg every 8 hours IV and methylprednisolone 30 mg every 12 hours IV. His dyspnea worsened, and he developed chest tightness. He died on day 11 from sepsis. The death was attributed to immunosuppression, and it was unrelated to the study drug.

The second death occurred in a male patient in his 60s with stage 4 adenosquamous carcinoma of the colon with metastases to the liver and peritoneum. On cycle 2, day 15, the patient was admitted to the hospital after a syncopal episode. At the time of admission, he was hypotensive, jaundiced, and lethargic. Laboratory tests demonstrated leukocytosis (WBC 22.07 × 10^9^/L, ANC 16.98 × 10^9^/L), the creatinine level was 3.8 mg/dL (normal, 0.7-1.2), and the bilirubin level was 21.4 mg/dL (normal values, 0.2-1.3 mg/dL). CT scan of the abdomen and pelvis showed diffuse hepatic metastases, without intrahepatic bile duct dilation, and diffuse wall thickening of the colon. He died 3 days later. His death was attributed to advanced cancer and sepsis, and it was deemed unrelated to the study drug.

### Anti-drug antibodies

Among 37 evaluable subjects (12 subjects in Part A and 25 subjects in Part B, defined as individuals who received at least 1 treatment cycle and had at least 1 post‑dose ADA sample collected), treatment‑induced ADA was observed in 2 of 12 subjects (16.7%) in Part A and in 10 of 25 subjects (40.0%) in Part B, resulting in an overall incidence of 12 of 37 subjects (32.4%). The onset of ADA induction varied across subjects, most occurring after 2 cycles of treatments. Except for 1 persistent high‑titer case in part A, all ADA‑positive subjects exhibited transient ADA responses with low titers. Although ADA‑positive subjects also showed neutralizing antibody (Nab) positivity, overall, the transient and low‑titer nature of both ADA and NAb responses indicates a low immunogenicity risk and suggests no meaningful impact on PK.

## PK assessments for OBI-999, total antibody, and MMAE

**Table:** 

PK Analytes	Cohorts	Part A dose escalation				Part B cohort expansion			Pooled RP2D*
		0.4 mg/kg	0.8 mg/kg	1.2 mg/kg	1.6 mg/kg	Pancreatic	Colorectal	Basket	
	Dose (mg/kg)	0.4	0.8	1.2	1.6	1.2	1.2	1.2	1.2
	*N*	3	3	6	3	5	6	4	21
**OBI-999**	*T*_max_ (day)	0.0639 (0.0632-0.0646)	0.0625 (0.0625-0.122)	0.0639 (0.0521-0.0694)	0.0590 (0.0590-0.0604)	0.0653 (0.0618-0.200)	0.0622 (0.0521-0.0653)	0.0628 (0.0569-0.0694)	0.0632 (0.0521-0.200)
	*C*_max_ (µg/mL)	5.87 (11.1)	11.3 (20.8)	20.6 (13.4)	33.7 (13.8)	18.3 (37.3)	22.4 (13.4)	23.9 (34.0)	21.2 (24.7)
	AUC_inf_ (day x µg/mL)	6.48 (12.3)	17.4 (40.9)	30.3 (17.6)	56.7 (26.4)	26.4 (25.9)	29.0 (26.6)	37.4 (51.5)	30.4 (33.2)
	T_1/2_ (day)	1.84 (36.5)	3.79 (39.4)	3.51 (37.1)	2.37 (15.3)	4.17 (6.19)	2.57 (34.2)	3.60 (11.0)	3.42 (29.6)
	CL (L/day)	4.84 (45.1)	3.81 (44.1)	2.73 (25.1)	2.67 (18.8)	2.59 (14.5)	3.09 (30.1)	2.79 (38.5)	2.81 (26.9)
	Vd (L)	14.3 (65.0)	18.4 (5.11)	14.2 (53.3)	9.03 (17.5)	15.7 (19.2)	10.7 (25.7)	14.1 (31.1)	13.5 (36.7)
**Total antibody**	T_max_ (day)	0.0639 (0.0632-0.0646)	0.0625 (0.0486-0.0625)	0.0639 (0.0521-0.0694)	0.0590 (0.0590-0.0604)	0.0646 (0.0563-0.0681)	0.0622 (0.0521-0.0653)	0.0628 (0.0569-0.0694)	0.0632 (0.0521-0.0694)
	*C*_max_ (µg/mL)	3.04 (6.97)	6.70 (19.6)	12.1 (23.5)	22.3 (21.6)	12.8 (41.9)	16.5 (31.2)	18.8 (43.1)	14.8 (38.0)
	AUC_inf_ (day x µg/mL)	3.17 (4.24)	10.0 (41.0)	18.9 (21.6)	37.6 (31.1)	22.1 (23.5)	25.4 (33.8)	30.9 (52.7)	23.8 (39.1)
	T_1/2_ (day)	0.829 (16.7)	1.46 (52.1)	1.80 (42.8)	1.90 (36.4)	1.98 (23.5)	1.98 (69.3)	2.42 (71.1)	2.01 (53.4)
	CL (L/day)	9.61 (38.9)	6.60 (44.3)	4.36 (20.7)	4.16 (33.2)	3.13 (25.7)	3.66 (36.6)	3.44 (43.1)	3.69 (31.3)
	Vd (L)	11.4 (42.0)	12.0 (18.3)	11.0 (41.0)	10.5 (10.6)	9.24 (46.9)	9.05 (39.7)	9.93 (39.7)	9.83 (39.6)
**MMAE**	*T*_max_ (day)	1.04 (0.373-1.07)	0.372 (0.365-0.375)	0.693 (0.365-1.06)	0.971 (0.375-6.03)	0.842 (0.290-3.05)	1.00 (0.242-1.06)	0.297 (0.198-1.04)	0.842 (0.198-3.05)
	*C*_max_ (ng/mL)	2.69 (54.5)	3.95 (99.2)	4.59 (56.3)	7.58 (78.7)	3.03 (68.2)	9.95 (59.1)	4.83 (29.1)	5.79 (75.6)
	AUC_inf_ (day x ng/mL)	13.9 (75.2)	18.1 (72.2)	26.4 (84.8)	62.6 (78.3)	23.3 (102)	54.7 (59.9)	26.2 (20.3)	32.7 (78.2)
	*T*_1/2_ (day)	2.78 (33.3)	3.28 (10.3)	3.21 (8.87)	4.18 (22.4)	3.06 (9.78)	3.31 (27.3)	3.00 (9.92)	3.16 (15.6)

Values indicate mean (CV%) except for *T*_max_, using median (range) values. *T*_max_, time to reach maximum concentration; *C*_max_, maximum observed concentration; AUC_inf_, area under the concentration-time profile extrapolating to time of infinity; *T*_1/2_, apparent terminal half-life; CL, clearance; Vd, apparent volume of distribution. *Data represent pooled Part A and Part B cohorts treated at 1.2 mg/kg

**Figure 6.**
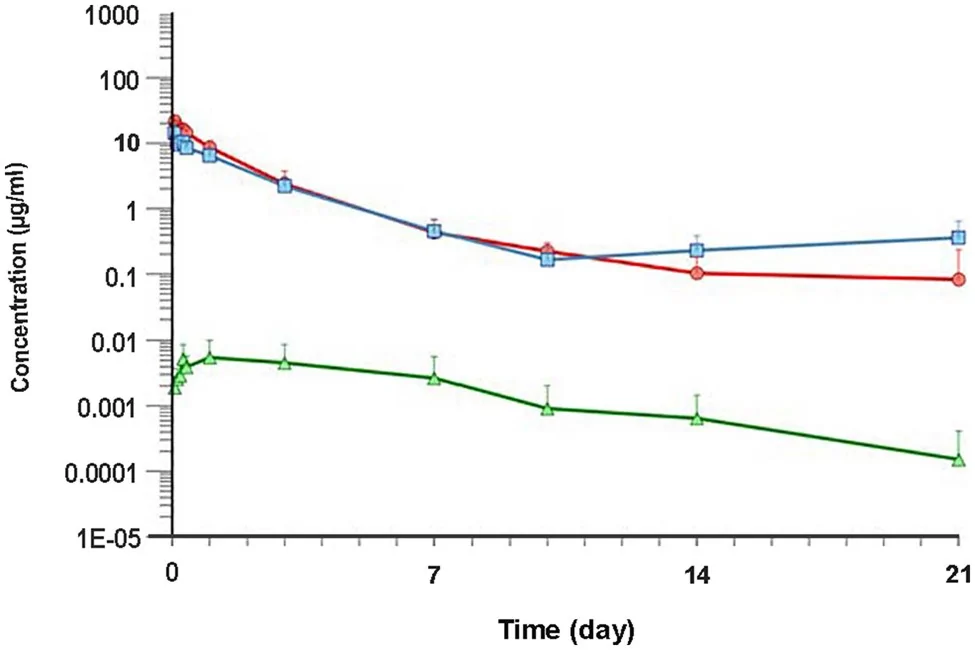
Mean concentration–time profiles of total antibody. OBI-999 (ADC) and unconjugated MMAE (MMAE) during cycle 1 of OBI-999 at RP2D (1.2 mg/kg, *n* = 34). One patient in Part B was excluded from the pharmacokinetic profile analysis due to pre-dose concentrations exceeding 5% of the corresponding *C*_max_. Abbreviations: ADC, antibody–drug conjugate; MMAE, monomethyl auristatin E; RP2D, recommended phase 2 dose.

**Notes:** PK assessments were conducted in both the dose‑escalation (Part A) and cohort‑expansion (Part B) phases (Supplementary Material). In Part A of the dose-escalation study, OBI-999 exhibited nonlinear PKs from 0.4 to 1.2 mg/kg, with lower clearance at higher doses. Following the first infusion of OBI‑999 at RP2D (1.2 mg/kg every 21 days), mean concentration–time profiles for total antibody (TAb), intact OBI‑999 (ADC), and unconjugated MMAE are presented in Figure 6 (data pooled from Part A [*n* = 6] and Part B [*n* = 28]). Peak serum concentrations of OBI‑999 and TAb occurred immediately at the end of infusion, followed by parallel declines, with both analytes remaining detectable at later time points.

Mean PK parameters after cycle 1 are summarized in the table. In Part B, intensive PK sampling was performed in the first 4 to 6 participants within each cohort, and only these participants were included in the PK parameter analyses. Systemic exposures of OBI-999 at the RP2D (1.2 mg/kg) were comparable across Part A and the various cohorts in Part B, with half-lives ranging from 2.57 to 4.17 days. For unconjugated MMAE, participants with colorectal cancer exhibited relatively higher systemic exposure as reflected by increased *C*_max_ and AUC_inf_ values. Although *T*_1/2_ of OBI-999 was slightly longer in pancreatic cancer, its clearance was comparable to that in other cohorts. Given the very limited sample size (*n* = 5), this observed difference in half-life was not considered clinically meaningful.

## Discussion

This is the first study that evaluated the efficacy of OBI-999 in patients with tumors with a Globo H H-score of ≥100. Disease stabilization was noted in 8 (34.8%) of 23 patients who were evaluable for response. Overall, 4 of 7 evaluable patients in the pancreatic cancer cohort, 2 of 8 patients in the colorectal cancer cohort, and 2 of 8 patients in the basket cohort (1 with cancer of the thymus and 1 with peritoneal cancer) had SD. The remaining patients had progressive disease. No objective response was noted. The median time on treatment was 22 days (range, 1-254 days). Despite promising preclinical findings showing tumor growth inhibition,^17^ this first-in-human phase 1/2 study did not demonstrate an objective response with OBI-999 at 1.2 mg/kg across the 3 cohorts evaluated (pancreatic, colorectal, and basket), and, therefore, according to the study design it was terminated.^18^

Overall, treatment with OBI-999 at 1.2 mg/kg was associated with a favorable safety and tolerability profile but a lack of robust response; these observations are consistent with the previously reported findings from a phase 1/2 study that evaluated OBI-888 as a monoclonal antibody treatment for solid tumors and with those of the Part A dose-escalation phase of the current study with OBI-999.^1^^,^^2^ Similar findings were also observed in an earlier randomized, double-blind, placebo-controlled, phase 2 trial assessing the efficacy and safety of a Globo H-KLH vaccine (adagloxad simolenin [OBI-822]/OBI-821) in metastatic breast cancer. In that study, OBI-822/OBI-821 was well tolerated but did not improve PFS.^11^ Notably, none of the patients in the OBI-999 1.2 mg/kg cohort in the Part A dose-escalation phase had grade 3/4 neutropenia or anemia, which are often seen with other antimicrotubule agents,^19^ and this factored into the choice of this dose for the Part B expansion.

Several factors may have contributed to the limited efficacy observed in this and other trials of Globo H–targeting therapies, including the challenge of impacting disease progression using a single-target therapy within the complex interplay of numerous biological signals that contribute to tumor growth.^2^ Combination strategies with Globo H-targeting agents and/or other antigen-focused immunotherapies alongside chemotherapy or other treatment options are likely needed to optimize tumor response. Recent preclinical findings in xenograft models have shown that OBI-999 exhibits a synergistic antitumor effect when combined with pembrolizumab.^20^

Although the overall incidence (Part A + B) of treatment‑induced ADA was high (32.4%), these transient, low‑titer responses suggest low immunogenicity risk with no PK impact; therefore, the impact of ADA on efficacy appears to be limited. In general, ADCs utilizing the same payload often achieve similar MTDs,^21^ as their dose‑limiting toxicities are primarily driven by systemic exposure to the released payload. When comparing OBI‑999 with other MMAE‑based ADCs, the MTD of OBI‑999 (1.2 mg/kg Q3W) is notably lower than that of many MMAE ADCs, which commonly reach 1.8-2.5 mg/kg.^21^ This lower tolerated dose may restrict the achievable systemic exposure of OBI-999, potentially resulting in insufficient exposure to achieve optimal antitumor efficacy. Notably, the lack of efficacy may in part be related to the MMAE payload in selected patients. A total of 7 patients had received nab-paclitaxel or docetaxel treatment; and all these patients had PD.

In vitro studies using differentiating neutrophils showed that OBI‑999 exhibited greater cytotoxicity than a benchmark MMAE ADC (Adcetris; data not shown). Since differentiating neutrophils do not express Globo H, and FcγR blockade reduced this cytotoxicity in a concentration‑dependent manner, Fc‑mediated, target‑independent mechanisms may play a role in OBI-999-associated neutropenia.^22^

Other contributing factors include antigen binding and internalization. The affinity of OBI‑999 to Globo H (approximately 10^−7^ M) is notably lower than the affinity typically observed for ADCs targeting membrane proteins such as HER2 or TROP2, which are often in the low‑nanomolar range. This lower binding strength may limit target engagement, and further optimization of Globo H–targeting antibodies may therefore be required to achieve improved efficacy.

Although all patients in the current study were required to have a Globo H score of at least 100, there was no indication that higher scores were predictive of disease control. This finding may be attributed to the heterogeneity of antigen expression, including Globo H expression, across different metastatic sites, and highlights the challenges of using antigen expression to identify patient subpopulations with the highest likelihood of clinical benefit.^23^

Some limited antitumor activity of OBI-999 was observed in this trial. It is notable that half of the patients achieving SD (*n* = 4) were in the pancreatic cancer cohort, including 3 patients who received treatment for at least 3 months (92, 157, and 254 days). Pancreatic cancer is often resistant to conventional therapies,^24^ and new treatment options are urgently needed. Globo H is overexpressed in approximately half of pancreatic tumors, and preclinical findings with OBI-999 showed excellent dose-dependent tumor growth inhibition in pancreatic xenograft models.^1^^,^^17^ No shared features among the patients with pancreatic cancer who had disease stabilization could be identified; all had undergone prior surgery and chemotherapy, the number of OBI-999 treatment cycles ranged from 2 to 13, and Globo H scores were between 150 and 282.

The decrease in tumor size for the patient with thymus cancer (thymic carcinoma with squamous cell differentiation) was markedly more pronounced than for any of the other tumors evaluated in this study, though PR was not achieved. The patient (male, in his 60s) received 9 cycles of OBI-999, which was the highest number of cycles among treated patients, and had previously undergone surgical intervention, radiation therapy, and chemotherapy. The reported Globo H score for this patient (145) was not predictive of the magnitude of tumor reduction, and other contributing factors are unknown. It is possible that an immunologic component was in effect, given the potential for “immunologically hot” thymus cancers and the possible role of Globo H blockade in enhancing anti-tumor immunity.^25^

In conclusion, OBI-999 was well tolerated and had a favorable safety profile but demonstrated limited clinical efficacy in patients with advanced solid tumors. This study was terminated early due to a lack of objective response. These findings highlight the challenges of targeting Globo H with a single-agent ADC and suggest that future investigations may require combination strategies, improved ADC design, or better patient selection based on tumor biology.

## Supplementary Material

oyag249_Supplementary_Data

## Data Availability

The data supporting the findings of this study are available within the main text and Supplementary Materials. All requests for further data sharing will be reviewed by MD Anderson and the study sponsors to determine whether they are subject to any intellectual property or confidentiality obligations. For further questions, please contact the corresponding author at atsimber@mdanderson.org.
