# Supplementary material for: Dose-expansion study of OBI-999, a Globo H-targeting antibody-drug conjugate, in patients with advanced solid tumors
Source: Oncologist. 2026 Jun 29;31(9):oyag249. doi: 10.1093/oncolo/oyag249 (PMC13503030; doi:10.1093/oncolo/oyag249)
Supplement: Supplementary file 1 [file oyag249_supplementary_data.zip › 061926_OBI999 supplementary material.docx]

**Supplemental Materials**

**Supplemental Methods**

*Eligibility Criteria*

Inclusion Criteria:

Patients must have met all of the following criteria in order to be included in the study (for both Parts A and B unless specified otherwise):

1. Male or female patients, 18 years of age or older at the time of consent.

2. Provide written informed consent prior to performing any study-related procedure.

3. Histologically or cytologically confirmed patients with advanced solid tumors.

4. Patients must have been treated with established standard-of-care therapy, or physicians have determined that such established therapy is not sufficiently efficacious, or patients have declined to receive standard-of-care therapy. In the latter case, the informed consent must state the effective therapies the patient is declining.

5. Measurable disease (i.e., at least one measurable lesion per Response Evaluation Criteria in Solid Tumors version 1.1 [RECIST 1.1]).

6. Eastern Cooperative Oncology Group (ECOG) performance status of 0 or 1.

7. Adequate organ function defined as:

a. Hepatic

i. Serum alanine aminotransferase (ALT) ≤3 × upper limit of normal (ULN), ≤5 × ULN in the presence of liver metastases

ii. Serum aspartate aminotransferase (AST) ≤3 × ULN, ≤5 × ULN in presence of liver metastases

iii. Serum bilirubin ≤1.5 × ULN (unless due to Gilbert’s syndrome or hemolysis)

b. Renal:

i. Creatinine clearance >50 mL/minute using Cockcroft Gault equation

c. Hematologic:

i. Absolute neutrophil count ≥1,500/μL

ii. Platelets ≥100,000/μL

iii. Hemoglobin ≥8 g/dL

8. Patient is willing and able to comply with all protocol-required assessments, visits, and procedures, including pretreatment tumor biopsy. Archival tumor biopsies are acceptable at baseline.

9. Females of childbearing potential must have negative serum pregnancy test prior to starting study therapy and agree to use a reliable form of contraceptive during the study treatment period and for at least 120 days following the last dose of study drug. Patient not of childbearing potential (i.e., permanently sterilized, postmenopausal) can be included in study. Postmenopausal is defined as 12 months with no menses without an alternative medical cause. Male patients must agree to use an adequate method of contraception during the study treatment period and for at least 120 days following the last dose of study drug.

10. Cannot be breast feeding.

11. Patients with human immunodeficiency virus (HIV) infection are eligible if CD4+ T-cell counts ≥ 350 cells/μL; patients on anti-retroviral therapy (ART) should be on an established dose for at least 4 weeks and have an HIV viral load less than 400 copies/mL prior to enrollment.

12. Patients with serological evidence of chronic hepatitis B virus (HBV) infection are eligible if they have an HBV viral load below the limit of quantification with or without concurrent viral suppressive therapy.

13. Patients with a history of hepatitis C virus (HCV) infection should have completed curative antiviral treatment and have a viral load below the limit of quantification.

14. Patients in Part B (Cohort-Expansion) must have documented Globo H H-score ≥ 100 from a qualified laboratory IHC assay in one of the following tumor types to be enrolled in the respective cohort:

- Cohort 1: Pancreatic cancer
  - Histopathologically confirmed patients with metastatic adenocarcinoma of the pancreas.
- Cohort 2: Colorectal cancer
  - Histopathologically confirmed patients with metastatic colorectal adenocarcinoma.
- Cohort 3: Basket (any solid tumor type other than those included in Cohorts 1 and 2)
  - Histologically or cytologically confirmed patients with advanced solid tumors.

Exclusion Criteria:

Patients meeting any of the following criteria are ineligible to participate in this study:

1. Less than 3 weeks from prior cytotoxic chemotherapy or radiation therapy; and less than 5 half-lives or 3 weeks, whichever is shorter, from prior biologic therapies, prior to the first dose of OBI-999.

2. Has undergone a major surgical procedure (as defined by the Investigator) or significant traumatic injury within 28 days prior to the first dose of OBI-999.

3. Sensory or motor neuropathy of Grade 2 or greater.

4. Patients with a history of solid organ transplant.

5. Unresolved toxicities from prior anticancer therapy, defined as having not resolved to Grade 0 or 1 (using National Cancer Institute Common Terminology Criteria for Adverse Events [NCI CTCAE] version 5.0), except for alopecia and laboratory values listed in the inclusion criteria.

6. Receipt of any prior therapy targeting Globo H.

7. Known hypersensitivity to OBI-999 or its excipients.

8. Has known untreated central nervous system (CNS) metastases. Patients with treated brain metastases are eligible if there is no evidence of progression for at least 4 weeks after CNS-directed treatment, as ascertained by clinical examination and brain imaging (magnetic resonance imaging [MRI] or computed tomography [CT]) during the screening period.

9. Has significant clinical cardiac abnormality (e.g., clinical heart failure or unstable angina).

10. Any medical co-morbidity that is life-threatening or, in the opinion of the Investigator, renders the patient unsuitable for participation in a clinical trial due to possible noncompliance, would place the patient at an unacceptable risk and/or potential to affect interpretation of results of the study.

11. Is receiving any concurrent prohibited medication

***Pharmacokinetics and Immunogenicity***

Pharmacokinetic (PK) assessments were conducted in both the dose-escalation (Part A) and cohort-expansion (Part B) phases. In Part B, intensive PK sampling was performed in the first 4 to 6 participants of each cohort. Serum PK samples for Cycle 1 and Cycle 2 were collected pre‑infusion on Day 1, at 30 minutes and 6 hours after the end of infusion on Day 1, and post‑infusion on Days 2, 4, 8, and 15. Serum concentrations of total antibody (TAb) and intact OBI-999 ADC were quantified using a validated enzyme-linked immunosorbent assay (ELISA) implemented on an electrochemiluminescence platform, with lower limits of quantitation (LLOQ) of 100 ng/mL and 15 ng/mL, respectively. Unconjugated monomethyl auristatin E (MMAE) levels were measured using a validated liquid chromatography-tandem mass spectrometry (LC-MS/MS) method with a sensitivity of 2 pg/mL. PK parameters were derived from participants who underwent intensive PK sampling, using actual time and doses by Phoenix WinNonlin software (version 8.3; Certara, Princeton, NJ, USA).

Immunogenicity was evaluated by assessing the presence of anti-drug antibodies (ADAs) against OBI-999 in serum samples using a tiered and validated electrochemiluminescence method, as previously described.^1^ Samples were collected pre‑infusion on Day 1 of Cycles 1–8, 12, and every 4 cycles thereafter in Part A and on Day 1 of Cycles 1-4, 8, and every 4 cycles thereafter in Part B, as well as at end‑of‑study/early‑termination. Samples that tested potentially positive in the screening tier were re-analyzed in a confirmatory assay (sensitivity level of 18.4 ng/mL for the screening assay and 43.0 ng/mL for the confirmatory assay). Only those samples confirmed positive were considered ADA-positive.
